# Increased expression of Polδ does not alter the canonical replication program *in vivo*

**DOI:** 10.12688/wellcomeopenres.16600.2

**Published:** 2021-05-04

**Authors:** Róbert Zach, Antony M. Carr

**Affiliations:** 1 Genome Damage and Stability Centre, School of Life Sciences, Science Park Road, University of Sussex, Falmer, Brighton, BN1 9RQ, UK

**Keywords:** Polymerase δ, over-expression, DNA replication, polymerase switch, polymerase usage sequencing, Schizosaccharomyces pombe, fission yeast

## Abstract

**Background:** *In vitro* experiments utilising the reconstituted *Saccharomyces cerevisiae* eukaryotic replisome indicated that the efficiency of the leading strand replication is impaired by a moderate increase in Polδ concentration. It was hypothesised that the slower rate of the leading strand synthesis characteristic for reactions containing two-fold and four-fold increased concentration of Polδ represented a consequence of a relatively rare event, during which Polδ stochastically outcompeted Polε and, in an inefficient manner, temporarily facilitated extension of the leading strand. Inspired by this observation, we aimed to determine whether similarly increased Polδ levels influence replication dynamics *in vivo* using the fission yeast *Schizosaccharomyces pombe* as a model system.

**Methods: **To generate *S. pombe* strains over-expressing Polδ, we utilised Cre-Lox mediated cassette exchange and integrated ****one or three extra genomic copies of all four Polδ genes. To estimate expression of respective Polδ genes in Polδ-overexpressing mutants, we measured relative transcript levels of *cdc1 ^+^*, *cdc6 ^+^* (or *cdc6 ^L591G^*), *cdc27 ^+^* and *cdm1 ^+^* by reverse transcription followed by quantitative PCR (RT-qPCR). To assess the impact of Polδ over-expression on cell physiology and replication dynamics, we used standard cell biology techniques and polymerase usage sequencing.

**Results: **We provide an evidence that two-fold and four-fold over-production of Polδ does not significantly alter growth rate, cellular morphology and S-phase duration. Polymerase usage sequencing analysis further indicates that increased Polδ expression does not change activities of Polδ, Polε and Polα at replication initiation sites and across replication termination zones. Additionally, we show that mutants over-expressing Polδ preserve WT-like distribution of replication origin efficiencies.

**Conclusions: **Our experiments do not disprove the existence of opportunistic polymerase switches; however, the data indicate that, if stochastic replacement of Polε for Polδ does occur i *n vivo*, it represents a rare phenomenon that does not significantly influence canonical replication program.

## Introduction

 Unchallenged duplication of the eukaryotic genome requires the coordinated action of three replicative polymerase complexes: Polα-primase (hereafter referred to as Polα), Polδ and Polε ( Burgers & Kunkel, 2017). According to the canonical model of eukaryotic replication, Polα and Polδ cooperate to discontinuously synthesise the lagging strand via the iterative production of short Okazaki fragments (OF), ca. 150bp, whereas Polε caries out continuous leading strand replication ( Clausen *et al.*, 2015; Daigaku *et al.*, 2015; Miyabe *et al.*, 2011). Interestingly, such strict division of labour does not always apply, and deviations have been documented ( Guilliam & Yeeles, 2020a).

 While polymerase activities of Polα and Polδ are indispensable for cell survival, the polymerase domain of Polε is not required for completion of replication in either *Saccharomyces cerevisiae* or *S. pombe* ( Feng & D’Urso, 2001; Kesti *et al.*, 1999). In both yeast experimental models it has been demonstrated that Polδ facilitates the leading strand synthesis when catalytically-inactive Polε is expressed ( Garbacz *et al.*, 2018; Miyabe *et al.*, 2015). Such findings have found support in *in vitro* experiments utilising reconstituted replisome system ( Yeeles *et al.*, 2017), confirming that, under certain circumstances, Polδ is competent in the leading strand replication.

 Indeed, it has been reported that Polδ replicates both DNA strands during homologous recombination restarted replication in *S. pombe* ( Miyabe *et al.*, 2015) and break induced replication in *S. cerevisiae* ( Donnianni *et al.*, 2019). Additionally, genomic analysis by polymerase usage sequencing (Pu-Seq) or HydEn-seq revealed that Polδ is involved in the initiation of the leading strand replication in unperturbed *S. cerevisiae* and *S. pombe* cells, respectively ( Daigaku *et al.*, 2015; Garbacz *et al.*, 2018; Zhou *et al.*, 2019). In agreement with such findings, PCNA-associated Polδ has been shown to play an important role in early stages of the leading strand replication *in vitro* ( Aria & Yeeles, 2018; Yeeles *et al.*, 2017). Moreover, it has recently been proposed that Polδ takes over the leading strand synthesis prior to replication fork termination ( Zhou *et al.*, 2019). The exact role of Polδ during the final stages of replisome progression is, however, yet to be clarified.

 Apart from homologous recombination dependent DNA synthesis and replication initiation, Polδ-mediated leading strand synthesis has been shown to occur in the context of polymerase uncoupling. It has been reported that cyclobutane pyrimidine dimer driven disengagement of CMG-associated Polε from the leading 3’OH generates a gap, the efficient filling of which requires the translesion synthesis machinery, as well as the action of Polδ ( Guilliam & Yeeles, 2020b). Additionally, it has been demonstrated that Polδ takes over the leading strand synthesis and performs an error-free bypass of oxidative DNA adducts thymine glycol and 8-oxoguanine ( Guilliam & Yeeles, 2021). In further support of a more generic function of Polδ in the leading strand synthesis, Polδ has been shown to proofread errors introduced by Polε in hyper mutator *pol2-M644G* mutants ( Bulock *et al.*, 2020). In line with all aforementioned observations, it has been shown that CMG-associated Polε exists in two mutually-exclusive conformations, of which only one facilitates DNA synthesis ( Zhou *et al.*, 2017).

 Intriguingly, according to *in vitro* studies of eukaryotic replication, two-fold and four-fold increase in Polδ concentration reduces the rate of the leading strand synthesis ( Yeeles *et al.*, 2017). It has been suggested that the observed retardation of leading strand replication represents a consequence of stochastic polymerase switching, during which Polδ outcompetes Polε and temporarily facilitates inefficient extension of the leading 3’ end. Since the effect of Polδ concentration on replisome progression and the hypothetical phenomenon of leading strand polymerase switching has not been investigated *in vivo*, we aimed to test whether similar a phenomenon manifests in living cells, potentially shedding light on a yet uncomprehended promiscuity of replicative polymerases.

## Methods

### Yeast culture and transformation

*S. pombe* cells were grown in yeast extract (YE) (Formedium, PCM0155) with supplements (Formedium, PSU0110) medium according to standard procedures ( Petersen & Russell, 2016). Briefly, cells (25% glycerol stocks stored at -80°C) were streaked onto an agar plate and incubated at 30°C for 2–3 days. Next, cells were inoculated into a liquid medium and cultivated at 30°C for ca. 36 h in the ISF-1-W shaker (Kuhner) with constant shaking (180 rpm). Cultures were diluted accordingly two times during the course of cultivation. Then appropriate amounts of cells were collected (depending on experiment) and processed further. Cells were transformed by the lithium-acetate based method ( Bähler *et al.*, 1998). Optical density (OD) of liquid cell cultures was assessed by WPA CO8000 Cell Density Meter (Biochrom). Doubling times were calculated using the formula: DT = 1/k, where DT stands for doubling time and k represents the slope of linear regression computed from a time-series of log _2_-transformed OD measurements. A list of strains used in this study is provided in Table 1.

**Table 1.** List of strains.

ID	Genotype	Origin
RZ42	*h- ade6-704 leu1-32 ura4-D18 I-5230932:[LoxP-cdc1-cdc27-ura4-cdc6-cdm1-LoxM3]*	This study
RZ47	*h- ade6-704 leu1-32*	Laboratory stock
RZ93	*h- ade6-704 leu1-32 ura4-D18 I-3325162:[LoxP-cdc1-cdc27-kanR-cdc6-cdm1-LoxM3] I-4734015:[LoxP-cdc1-cdc27-* *natR-cdc6-cdm1-LoxM3] I-5230932:[LoxP-cdc1-cdc27-ura4-cdc6-cdm1-LoxM3]*	This study
655	*h- ade6-? leu1-32 ura4-D18 rnh201::kanR cdc20M630F*	Laboratory stock
856	*h- ade6-704 leu1-32 ura4-D18 rnh201::kanR cdc6L591G*	Laboratory stock
1141	*h- ade6-704 leu1-32 ura4-D18 rnh201::kanR pol1L850F*	Laboratory stock
RZ57	*h- ade6-704 leu1-32 ura4-D18 I-5230932:[LoxP-cdc1-cdc27-ura4-cdc6L591G-cdm1-LoxM3] rnh201::hygR cdc6L591G*	This study
RZ62	*h- ade6-704 leu1-32 ura4-D18 I-5230932:[LoxP-cdc1-cdc27-ura4-cdc6-cdm1-LoxM3] rnh201::hygR cdc20M630F*	This study
RZ68	*h- ade6-704 leu1-32 ura4-D18 I-5230932:[LoxP-cdc1-cdc27-ura4-cdc6-cdm1-LoxM3] rnh201::hygR pol1L850F*	This study
RZ112	*h- ade6-704 leu1-32 ura4-D18 I-3325162:[LoxP-cdc1-cdc27-kanR-cdc6L591G-cdm1-LoxM3] I-4734015:[LoxP-cdc1-* *cdc27-natR-cdc6L591G-cdm1-LoxM3] I-5230932:[LoxP-cdc1-cdc27-ura4-cdc6L591G-cdm1-LoxM3] rnh201::hygR* *cdc6L591G*	This study
RZ116	*h- ade6-704 leu1-32 ura4-D18 I-3325162:[LoxP-cdc1-cdc27-kanR-cdc6-cdm1-LoxM3] I-4734015:[LoxP-cdc1-cdc27-* *natR-cdc6-cdm1-LoxM3] I-5230932:[LoxP-cdc1-cdc27-ura4-cdc6-cdm1-LoxM3] rnh201::hygR pol1L850F*	This study
RZ118	*h- ade6-704 leu1-32 ura4-D18 I-3325162:[LoxP-cdc1-cdc27-kanR-cdc6-cdm1-LoxM3] I-4734015:[LoxP-cdc1-cdc27-* *natR-cdc6-cdm1-LoxM3] I-5230932:[LoxP-cdc1-cdc27-ura4-cdc6-cdm1-LoxM3] rnh201::hygR cdc20M630F*	This study
RZ331	*h- ade6-704 leu1-32 cdc2asM17*	This study
RZ332	*h- ade6-704 leu1-32 cdc2asM17 I-5230932:[LoxP-cdc1-cdc27-ura4-cdc6-cdm1-LoxM3]*	This study
RZ333	*h- ade6-704 leu1-32 cdc2asM17 I-3325162:[LoxP-cdc1-cdc27-kanR-cdc6-cdm1-LoxM3] I-4734015:[LoxP-cdc1-cdc27-* *natR-cdc6-cdm1-LoxM3] I-5230932:[LoxP-cdc1-cdc27-ura4-cdc6-cdm1-LoxM3]*	This study

### Microscopy

1 mL of exponentially growing cells was centrifuged (1000 × g, 5 min, 25°C) and the cell pellet resuspended in 1 mL of 70% ethanol. 500 µL of fixed cells were collected by centrifugation (1000 × g, 5 min, 25°C) and re-suspended in 50 µL of H _2_O containing 1µM 4′,6-diamidino-2-phenylindole (DAPI). Cells were incubated at room temperature in the dark for at least 15 min, and then analysed by microscopy using a Nikon E400 system. Cell lengths were determined from DIC images by measuring the distance between the opposite poles of the cell using ImageJ software (version 1.51m9) ( Schneider *et al.*, 2012). At least 200 cells per sample were scored.

### Cell synchronisation and DNA content analysis

Exponentially growing *cdc2 ^asM17^* cells (OD _600_ = 0.1–0.2; 1–2 × 10 ^6^ cells/mL) were treated with 2µM 3-Br-PP1 (abcam, ab143756) for 3 h. A 50mL fraction 3-Br-PP1-treated culture was centrifuged (1000 × g, 5 min, 25°C) and the cell pellet washed with 50 mL of fresh YES medium pre-heated to 30°C. Washed cells were re-suspended in 50 mL of fresh pre-heated YES and incubated at 30°C. In 15-min intervals, 1mL aliquots of synchronous cell culture were centrifuged (1000 × g, 3 min, 25°C) and collected cells fixed in 1 mL of 70% ethanol. For each time point, 500 µL of fixed cells were centrifuged (1000 × g, 3 min, 25°C), the supernatant was discarded, and the cell pellet re-suspended in 500 µL of sodium citrate (50 mM, pH = 7) containing 1 mg/mL RNase A (NEB, T3018L). The resulting cell suspension was incubated for 3 h at 37°C, and then mixed with 500 µL of sodium citrate (50 mM, pH = 7) containing 2µM SYTOX Green (Invitrogen, S7020). Samples were analysed using an Accuri C6 flow cytometry system (Beckman Coulter). Data were analysed by BD CSampler software (version 1.0.264.21) and R (version 4.0.0) ( https://www.R-project.org; R Core Team, 2020).

### Cre-recombination mediated cassette exchange (RMCE)

Leucine-auxotrophic cells carrying one of three LoxP-LoxM3 integration sites (I-3325162:[LoxP-rts-ura4 ^+^], I-4734015:[LoxM3-kanR-LoxP], I-5230932:[LoxM3-kanR-LoxP]) were transformed with one of the Polδ Cre-Lox integration vectors (pRZ02, pRZ03, pRZ04, pRZ05, pRZ06, pRZ07) listed in Table 2. Leucine-prototrophic transformants (containing respective Polδ Cre-Lox integration vector) were selected on EMM plates lacking leucine. Single clones were selected and grown over-night in liquid YES medium. Next, leucine-auxotrophic colonies (lacking the transformed Polδ Cre-Lox vector) carrying a selection marker associated with respective Polδ integration events were selected ( Watson *et al*., 2008).

**Table 2.** List of plasmids.

ID	Annotation	Origin
pRZ02	pAW8_cdc1-cdc27-ura4-cdc6-cdm1	This study, derived from pAW8 ( Watson *et al.*, 2008)
pRZ03	pAW8_cdc1-cdc27-ura4-cdc6L591G-cdm1	
pRZ04	pAW8_cdc1-cdc27-natR-cdc6-cdm1	
pRZ05	pAW8_cdc1-cdc27-natR-cdc6L591G-cdm1	
pRZ06	pAW8_cdc1-cdc27-kanR-cdc6-cdm1	
pRZ07	pAW8_cdc1-cdc27-kanR-cdc6L591G-cdm1	

### Plasmids

Cre-Lox integration plasmids carrying 4 Polδ genes ( *cdc1 ^+^*, *cdc27 ^+^*, *cdm1 ^+^*, *cdc6 ^+^* or *cdc6 ^L591G^*) and one of the three selection markers (NatR, KanR, ura4 ^+^) were derived from the previously characterised pAW8 vector (Addgene, 110222) ( Watson *et al.*, 2008) by standard restriction insertion cloning. Briefly, insert DNA fragments ( *Sph*I- *cdc1 ^+^*- *Apa*I, *Apa*I- *cdc27 ^+^*- *Xho*I, *Xho*I- *ura4 ^+^*- *Sac*I, *Sac*I- *cdc6 ^+^*- *Sbf*I, *Sac*I- *cdc6 ^L591G^*- *Sbf*I, *Sbf*I- *cdm1 ^+^*- *Spe*I, *Apa*I- *cdc27 ^+^*-NatMX6- *Sac*I, *Apa*I- *cdc27 ^+^*-KanMX6- *Sac*I) were generated by high-fidelity PCR with KOD Hot Start DNA Polymerase (Merck Millipore, 71085–3) and purified with QIAquick PCR Purification Kit (QIAGEN, 28104) or QIAquick Gel Extraction Kit (QIAGEN, 28704). *Sac*I- *cdc6 ^L591G^*- *Sbf*I *, Apa*I- *cdc27 ^+^*-NatMX6- *Sac*I and *Apa*I- *cdc27 ^+^*-KanMX6- *Sac*I were produced by overlap extension PCR. Generated Polδ gene fragments contained intact 5’UTR and 3’UTR sequences, as well as upstream and downstream regions of 663–980 bp. pAW8 vector and insert DNA fragments were digested by respective restriction enzymes and ligated over-night at 18°C using T4 DNA ligase (NEB, M0202S). Each ligation reactions contained 50 ng of pAW8 vector and three-fold molar excess of respective DNA fragments. Ligation reactions were incubated in T3 Thermocycler (Biometra). Restriction digestion reactions were performed according to manufacturer’s instructions with the following restriction enzymes: *Sph*I-HF (NEB, R3182S); *Apa*I (NEB, R0114S); *Xho*I (NEB, R0146S); *Sac*I-HF (NEB, R3156S); *Spe*I-HF (NEB, R3133S); *Sbf*I-HF (NEB, R3642S); *Sal*I-HF (NEB, R3138S); *Bam*HI-HF (NEB, R3136S). Ligation products were transformed into DH5-Alpha *E. coli* competent cells. Plasmids were purified using a QIAprep Spin Miniprep Kit (QIAGEN, 27104). A list of generated plasmids is provided in Table 2.

### RT-qPCR

Total RNA was isolated from 1–2 mL of exponentially growing cells (OD _600_ = 0.5; 5×10 ^6^ cells/mL) using MasterPure Yeast RNA purification kit (Cambio Ltd, MPY03100). RNA was converted to cDNA utilising a RevertAid First Strand cDNA Synthesis Kit (ThermoFisher Scientific, K1621) and random hexamer primers. Relative transcript levels were determined by qPCR with Luna Universal qPCR Master Mix (NEB, M3003E) and an AriaMx Real-time PCR System (Agilent Technologies). qPCR reactions were prepared by mixing 10 µL of 2× Luna Universal qPCR Master Mix, 0.5 µL of forward and reverse primers (10 µM), 2 µL of 1000-fold diluted cDNA and 7 µL of nuclease-free H _2_O. Thermal cycling conditions were: Hot Start: 95°C for 3 min; Cycling (45×): 95°C for 15 s, 60°C for 30 s. Relative transcript levels were calculated using the equation RNA _target_ = 2 ^-Cq (target)^ / 2 ^-Cq (reference)^, where RNA _target_ represents the relative transcript level of a given target gene, and Cq (target) and Cq (reference) stand for PCR cycle quantification values of target and reference genes, respectively. *act1* was used as the reference gene. A list of qPCR primers (obtained from Integrated DNA Technologies) used in this study is provided in Table 3.

**Table 3.** List of qPCR primers.

ID	Sequence (5’ – 3’)	Target	Origin
RZ67	CAACTATCCTTCCTCAACAG	*cdc1* (134 bp)	This study
RZ68	GCTAGTAGCCAACACAAAATG		
RZ69	CGTTCACGATTCTGAAGATG	*cdc27* (102 bp)	This study
RZ70	ATAATTTCCTGAGGTTCGTC		
RZ75	CCTGCAATAAATCCTGAGAAG	*cdc6* (109 bp)	This study
RZ76	CATTGTCAGTAACACCAAAC		
RZ81	TTCATTCTAGTACCGCAGTG	*cdm1* (142 bp)	This study
RZ82	TGTGGGATTGACTTGAATTAC		
RZ87	TCCTCATGCTATCATGCGTCTT	*act1* (78 bp)	( Převorovský *et al.*, 2016)
RZ88	CCACGCTCCATGAGAATCTTC		

### Pu-Seq library preparation

For all strains presented, two sets of Pu-Seq libraries were prepared. One set was prepared as described previously ( Daigaku *et al.*, 2015; Keszthelyi *et al.*, 2015). Briefly, 10–20 µg of genomic DNA containing increased quantities of misincorporated ribonucleotides (rNMPs) was treated with 0.3M NaOH for 2 h at 55°C. Digested DNA was run on a 2% agarose gel (in 0.5× TBE). 300–500bp ssDNA fragments were gel extracted and subjected to complementary second strand synthesis primed by random 8-mers (obtained from Integrated DNA technologies). Resulting double-stranded (dsDNA) fragments were converted to Illumina sequencing libraries using NEBNext Ultra DNA library prep kit for Illumina (NEB, E7645S) and NEBNext multiplex oligos for Illumina (NEB, E7335). The second set of Pu-Seq libraries was prepared according to a modified version of the established GLOE-Seq protocol ( Sriramachandran *et al.*, 2020), which utilises two subsequent ligations of adapter/splinter oligonucleotides, first to rNMP-dependent phosphorylated 5’ ends and, following sonication, to 3’ ends of the ssDNA fragments. rNMP-dependent 5' ends were generated from genomic DNA containing increased quantities of misincorporated ribonucleotides treated by RNAse H2 (NEB, M0288S) and subsequently denatured at 95°C. Sequencing was performed on an Illumina NextSeq 500 sequencer. Sequencing reads were mapped onto the reference genome using Bowtie2 ( Langmead & Salzberg, 2012).

### Pu-Seq data analysis

Polymerase tracks at any given 300bp bin were calculated using the equation PT = (PT = (RT – RB) / (RT + RB), where PT represents polymerase track, and R _T_ and R _B_ stand for rNMPs mapped to the top and the bottom DNA strands, respectively. Polymerase tracks were determined for each biological repeat separately, then averages of the two repeats were used for subsequent analysis. Positions and efficiencies of origins of replication were determined from differential values of polymerase tracks, similarly to ( Daigaku *et al.* (2015). Briefly, for all three datasets (Polδ, Polε, Polα), the difference of each neighbouring datapoint of polymerase track values (smoothed by simple moving average of 3) was calculated as Diff _i_ = PT _i_ – PT _i-1_, where Diff _i_ represents differential value at position i, and PT _i_ – PT _i-1_ stand for smoothed polymerase track values at positions i and i-1, respectively. Differential value of the first bin on a given chromosome was assigned 0. Polε differentials and the opposites of Polδ and Polα differentials were averaged and smoothed by simple moving average of 3. Then, positive peaks (indicating sharp inclinations in the data) were selected. Differential peaks containing two or more distinct maxima separated by at least four bins were treated as independent peaks. Peaks with maxima bellow 30 ^th^ percentile were disregarded. Each independent differential peak represented an origin of replication, the efficiency of which was estimated as 50% of the sum of its values. 259 replication initiation regions and 147 termination zones were selected using wild-type (WT) origin efficiency data. For comparison purposes, origin efficiencies were normalised assuming that the efficiency of the most efficient origin was 100%. Data were analysed in R ( https://www.R-project.org; R Core Team, 2020) using a custom script (see *Software availability*).

## Results

### Brief overview of polymerase usage sequencing

**Figure 1.**
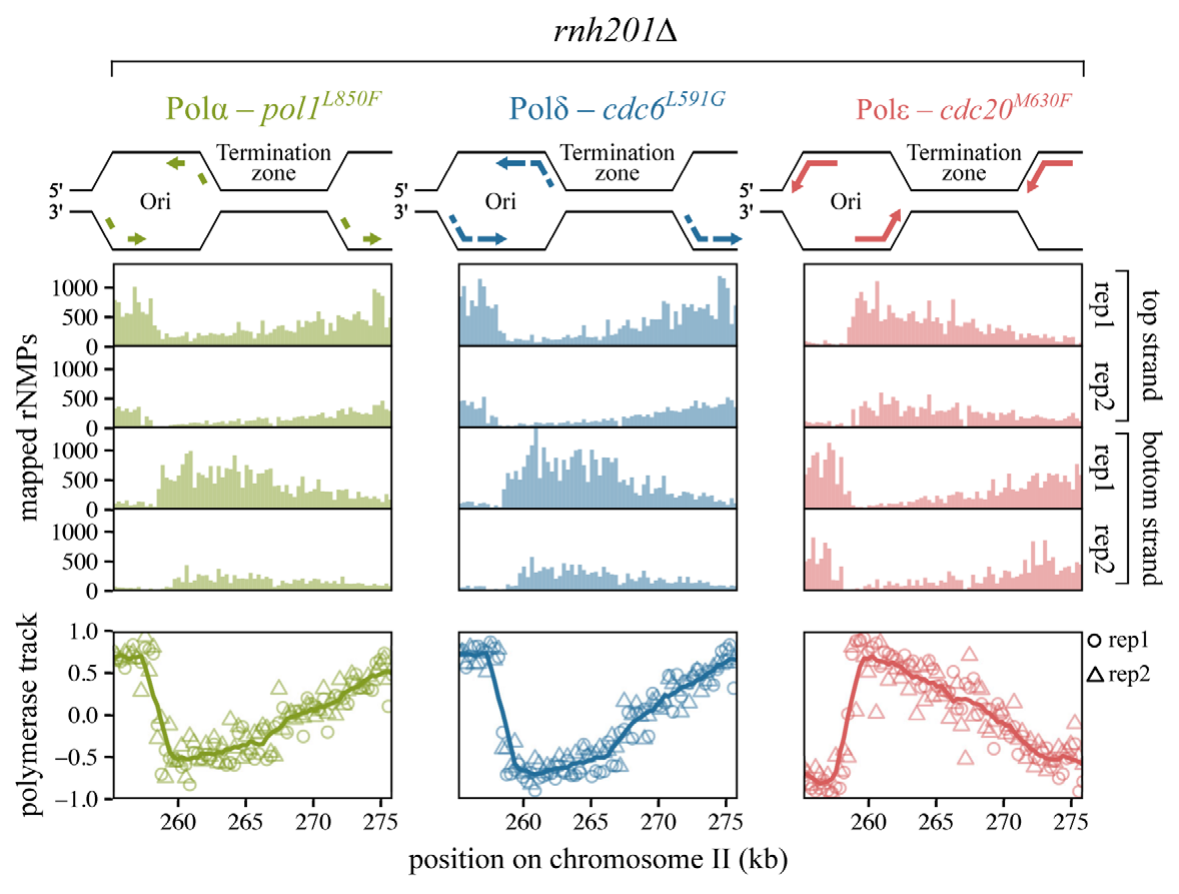
General Pu-Seq analysis. Top panel – cartoon representation of Polα, Polδ and Polε activities around an origin of replication (Ori) and across adjacent termination zone. Respective polymerase mutations employed in Pu-Seq are indicated. Middle panel – Example of genomic ribonucleotides (rNMPs; presented as 300bp bins) detected by Pu-Seq in *rnh201∆* cells expressing Cdc6 ^L591G^, Cdc20 ^M630F^ and Pol1 ^L850F^. A representative locus adjacent to an origin of replication is shown. Bottom panel – Polymerase tracks calculated for Polα, Polδ and Polε at the representative locus. For each polymerase, polymerase tracks are calculated from rNMPs mapped to the top and the bottom DNA strands as: PT = (RT – RB) / (RT + RB), where PT represents polymerase track, and R _T_ and R _B_ stand for rNMPs mapped to the top and the bottom DNA strands, respectively. Positive and negative values indicate predominant polymerase activity on the top and the bottom DNA strands, respectively. Data from 2 independent experiments are shown.

 Pu-Seq methodology determines the genome-wide polymerase activities by detecting the traces of rNMPs misincorporated by mutated Polδ ( *cdc6 ^L591G^*), Polε ( *cdc20 ^M630F^*) or Polα ( *pol1 ^L850F^*) ( Daigaku *et al.*, 2015; Keszthelyi *et al.*, 2015). In Pu-Seq, respective polymerase mutant strains also carry a deletion of *rnh201*, the catalytic subunit of RNase H2 complex, disruption of which abrogates ribonucleotide excision repair (RER) and thus stabilises misincorporated rNMPs ( Daigaku *et al.*, 2015). To assess activities of individual replicative polymerases, we employed a strategy previously used to analyse Okazaki fragment sequencing data ( Petryk *et al.*, 2016). Briefly, activities of Polδ, Polε and Polα at any given locus are expressed as polymerase tracks, which are proportional differences of rNMPs misincorporated in the top and the bottom DNA strands ( Figure 1).

### Construction and characterisation of Polδ-overexpressing strains

**Figure 2.**
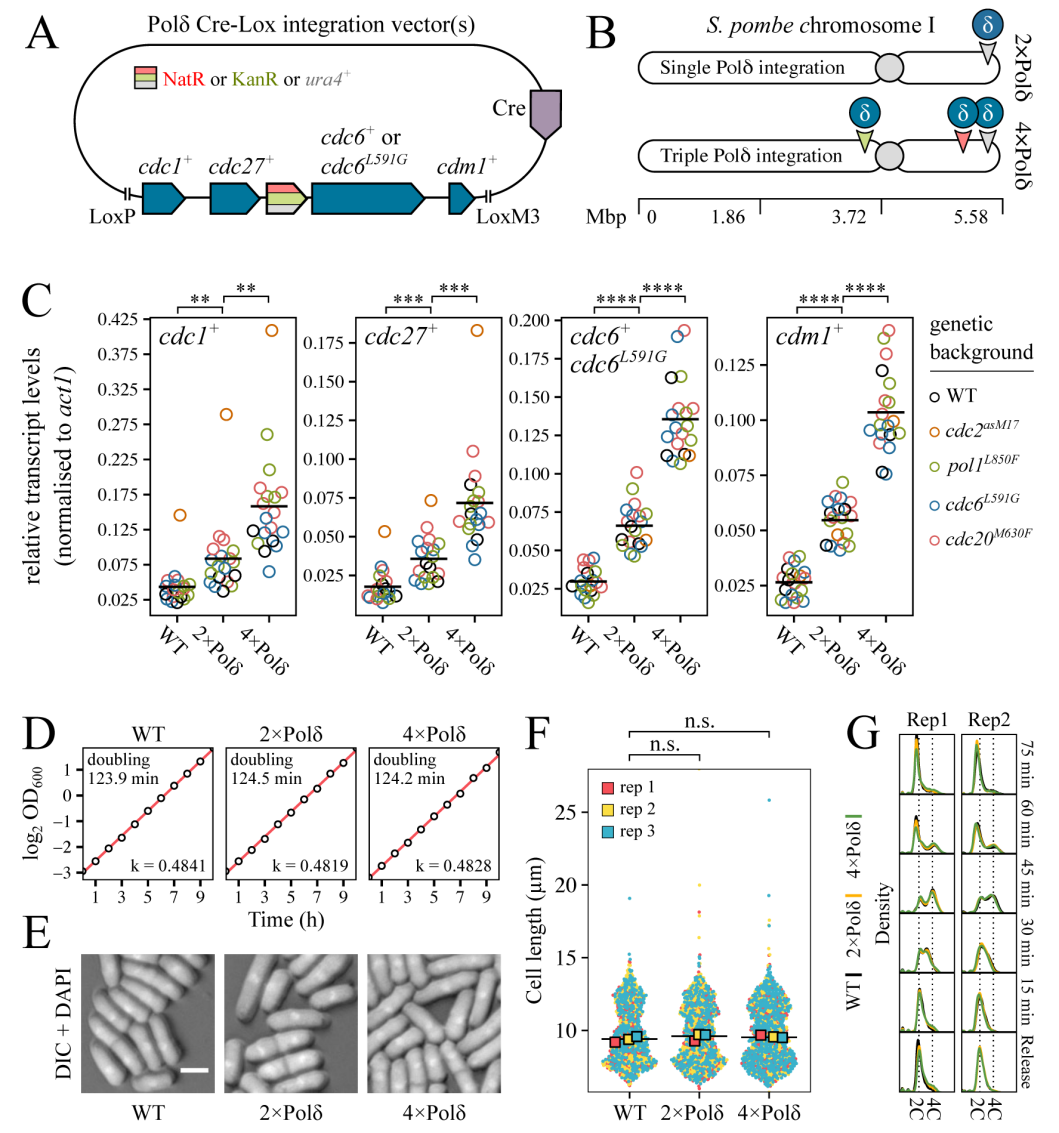
Construction and characterisation of mutants over-expressing Polδ. ( **A**) Simplified map of Cre-Lox vector(s) that were used to integrate extra copies of Polδ genes. Each vector carries genes constituting wild-type (WT) or L591G-mutated Polδ holoenzyme and one of three selection markers: NatR or KanR or *ura4 ^+^*. Cre – Cre recombinase ( **B**) Graphical representation of genomic Polδ integration site(s) in 2×Polδ and 4×Polδ cells. ( **C**) Relative transcript levels of Polδ genes ( *cdc1 ^+^*, *cdc27 ^+^*, *cdc6 ^+^/cdc6 ^L591G^* and *cdm1 ^+^*) in the indicated mutants measured by RT-qPCR. Mutants designated as *pol1 ^L850F^*, *cdc6 ^L591G^* and *cdc20 ^M630F^* also carried *rnh201∆*. Individual points represent data from independent experiments. For WT, 2×Polδ and 4×Polδ cells, 19 independent measurements were taken (all genetic backgrounds combined). Horizontal lines represent means. Statistical significance was determined by the unpaired two-sample t-test. ** p ≤ 0.01; *** p ≤ 0.001;**** p ≤ 0.0001 ( **D**) Representative growth curves of WT, 2×Polδ and 4×Polδ cells. Optical density (OD) was measured in 1h intervals for total 10 h. Time-series of log _2_-transformed OD measurements are presented. Red lines represent linear regression models. Slopes of linear regression models (k) and calculated doubling times are indicated. ( **E**) Representative images of WT, 2×Polδ and 4×Polδ cells stained with DAPI. Composite images of DIC and DAPI channels are shown. Scale bar represents 5 µm. ( **F**)Distributions of cell lengths of WT, 2×Polδ and 4×Polδ cells. Data from three independent experiments are shown. Squares represent medians of individual experiments. Horizontal line represents the median of merged data. Statistical significance was determined by the unpaired two-sample Wilcoxon test. n.s. = not significant. ( **G**)DNA profiles of WT, 2×Polδ and 4×Polδ cells synchronised in G2. Results from two independent experiments are shown.

 To achieve an approximate two-fold and four-fold upregulation of the whole Polδ complex, we aimed to increase the genomic copy number of all four Polδ genes. We constructed a set of Cre-Lox integration vectors, each of which carried a distinct selection marker (NatR, KanR, *ura4 ^+^*) and all four genes constituting either WT ( *cdc6 ^+^*, *cdc1 ^+^*, *cdc27 ^+^*, *cdm1 ^+^*) or L591G-mutated ( *cdc6 ^L591G^*, *cdc1 ^+^*, *cdc27 ^+^*, *cdm1 ^+^*) Polδ ( Figure 2A). Employing Cre-Lox recombination mediated cassette exchange ( Watson *et al.*, 2008), we generated three distinct Polδ genomic integrations and created strains carrying either one (2×Polδ) or three (4×Polδ) extra copies of either WT or L591G-mutated Polδ holoenzyme ( Figure 2B). Using WT Polδ integrations, we constructed 2×Polδ and 4×Polδ strains expressing Cdc2 ^asM17^ (Cdk1 variant inhibitable by the ATP analogue 3-Br-PP1), which allowed us to synchronise cells in G2 and assess their progression through the S-phase ( Aoi *et al.*, 2014). Additionally, we constructed 2×Polδ *rnh201∆* and 4×Polδ *rnh201∆* mutants expressing either Cdc20 ^M630F^ or Pol1 ^L850F^, which allowed us to determine whether the activities of Polε and Polα were altered in cells over-expressing Polδ. In a similar manner, utilising L591G-mutated Polδ integrations, we produced 2×Polδ *rnh201∆* and 4×Polδ *rnh201∆* mutants exclusively expressing Cdc6 ^L591G^, which allowed us to asses activity of Polδ at different expression levels.

 To validate that 2×Polδ and 4×Polδ mutants displayed increased expression of Polδ genes, we measured relative transcript levels of *cdc1 ^+^*, *cdc27 ^+^, cdc6 ^+^/cdc6 ^L591G^*, and *cdm1 ^+^* by RT-qPCR. In all genetic backgrounds tested, 2×Polδ and 4×Polδ mutants displayed a significant increase in relative transcript levels of all four Polδ genes ( Figure 2C). Unfortunately, due to the unavailability of commercial antibodies recognising Polδ subunits in *S. pombe*, we were unable to confirm that protein levels of the Polδ subunits were also increased. It has been previously reported, however, that plasmid-based over-expression of each of the four Polδ subunits is achievable in *S. pombe* ( Kang *et al.*, 2000; MacNeill *et al.*, 1996; Reynolds *et al.*, 1998). Consequently, we reasoned that elevation of Polδ transcript levels represented sufficient proof of *bona-fide* upregulation.

 To determine the fundamental cellular consequences of Polδ-overexpression, we assessed growth rate and cellular morphology of WT, 2×Polδ and 4×Polδ cells. Polδ-overexpressing mutants displayed WT-like growth parameters and did not develop any cellular or nuclear defects ( Figure 2D and 2E). Accordingly, increased Polδ expression did not alter the distribution of cell sizes ( Figure 2F). To assess whether increased Polδ expression influenced progression through S-phase specifically, we synchronised WT, 2×Polδ and 4×Polδ cells with a Cdc2 ^asM17^ background in G2 by the addition of 3-Br-PP1 and analysed changes in DNA content in 15-min intervals after release. Progression through S-phase in 2×Polδ and 4×Polδ mutants was undistinguishable from WT cells ( Figure 2F), suggesting that the over-production of Polδ did not change S-phase progression. Taken together, a moderate increase in Polδ expression did not have a notable impact on cell cycle or replication progression.

### Replication dynamics

**Figure 3.**
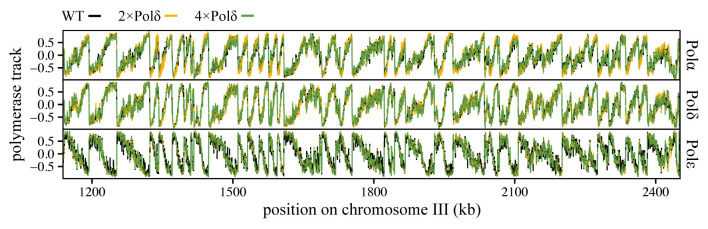
Representative polymerase tracks. Polδ, Polε and Polα tracks across the right arm of chromosome III in WT, 2×Polδ and 4×Polδ cells. Means of two independent experiments are shown.

**Figure 4.**
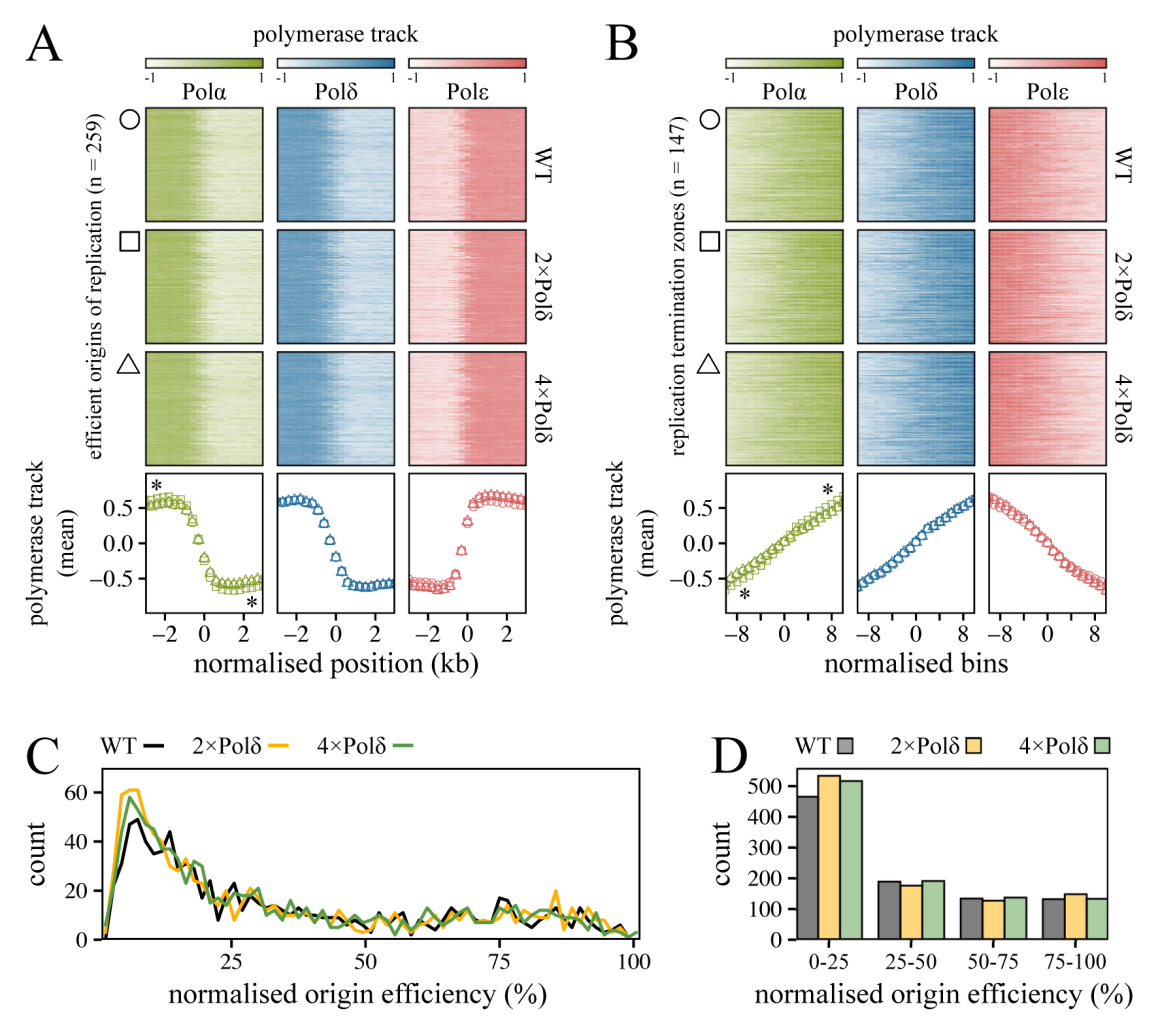
Pu-Seq analysis of mutants over-expressing Polδ. ( **A**, **B**) Polδ, Polε and Polα tracks around 259 efficient origins of replication ( **A**) and across 147 termination zones ( **B**). Individual regions and means are shown. Polδ expression levels are indicated: circles – wild-type (WT); squares – 2×Polδ; triangles – 4×Polδ. Chromosomal coordinates around efficient origins were centred relative to the position of an origin. Data constituting termination zones were equally binned, and bins were centred relative to the midpoint of a termination zone. * Minor deviations in Polα tracks ( **C**, **D**) Distribution of normalised origin efficiencies in WT, 2×Polδ and 4×Polδ cells. ( **A**– **D**) Means of two independent experiments were analysed.

 To investigate the potential influence of Polδ-overexpression on replication dynamics in greater detail, we performed two independent Pu-Seq experiments, each of which addressed activities of Polδ, Polε and Polα, in WT, 2×Polδ and 4×Polδ cells. Overall, in all genetic backgrounds tested, Polδ, Polε and Polα tracks displayed very little variation ( Figure 3), suggesting that increased Polδ levels did not dramatically alter the properties of replication. To capture a genome-wide view of replication, we examined regions around efficient origins of replication [characterised by estimated firing efficiency (Ori _Eff_) of at least 40%] and regions constituting replication termination zones, which were defined by two efficient origins (Ori _Eff_ > 40%) and did not contain any intermediary efficiency origins (20% < Ori _Eff_ < 40%). Analysis of Polδ and Polε tracks associated with 259 efficient origins and 147 termination zones did not reveal any notable differences ( Figure 4A and 4B). We observed that Polα tracks in 2×Polδ cells displayed marginal deviation from the WT profile ( Figure 4A and 4B); however, considering that the observed difference was not reflected in 4×Polδ cells, we concluded this observation represented a technical, rather than biological phenomenon. We reasoned that if increased Polδ levels negatively affected replisome progression, 2×Polδ and 4×Polδ mutants would be expected to display increased activity of low and intermediary efficiency origins. Polδ-overexpressing cells, however, retained a WT-like distribution of genome-wide origin efficiencies, which further indicated normal replication progression ( Figure 4C and 4D). Taken together, we concluded that, in our experimental system, a moderate increase in Polδ levels did not result in any observable changes in replication dynamics.

## Conclusions

 In this study, we tested whether a moderate (2–4-fold) increase in Polδ expression impairs, or in any way alters, replication dynamics under normal conditions in *S. pombe*. The presented experiments were inspired by report that a two-fold and four-fold increase in Polδ concentration reduces the rate of the leading strand synthesis *in vitro*, hypothesised to be due to stochastic polymerase switching, during which Polδ outcompetes Polε and temporarily facilitates the extension of the leading strand ( Yeeles *et al.*, 2017).

 We constructed a set of strains carrying either one or three extra copies of all Polδ genes and validated that these Polδ integrations resulted in increased transcription of the respective Polδ components: *cdc1 ^+^*, *cdc27 ^+^*, *cdc6 ^+^* and *cdm1 ^+^*. We were unable to explore if the Polδ subunits were upregulated at the protein level. However, considering that successful ectopic over-production of Polδ subunits has been reported in the seminal literature ( Kang *et al.*, 2000; MacNeill *et al.*, 1996; Reynolds *et al.*, 1998), we argue that our experimental design conveyed a genuine Polδ over-production.

 We determined that cells characterised by up to four-fold increased Polδ expression do not exhibit defects in growth and cell cycle progression. Furthermore, utilising Pu-Seq methodology, we demonstrated that genome-wide replication dynamics in 2×Polδ and 4×Polδ mutants is virtually indistinguishable from WT, arguing against the notion of stochastic polymerase switching or any other impairment of DNA replication induced by over-production of Polδ.

 Naturally, it is still possible that we simply did not reach the threshold of Polδ expression that is required for the polymerase-switch to occur at frequencies detectable by Pu-Seq. Higher cellular levels of Polδ could be achieved by ectopic or strong promoter-driven expression of Polδ genes; however, we argue that such an extensive Polδ over-production would constitute a non-physiological system, which would no longer be biologically relevant in relation to the reported *in vitro* data ( Yeeles *et al.*, 2017). Moreover, it has been shown that gross over-expression of *cdc6 ^+^* is detrimental to overall cell physiology ( MacNeill *et al.*, 1996), which would likely make Pu-Seq experiments difficult to interpret or impossible to carry out. We also argue that promoter manipulation or plasmid-based over-expression would disrupt the stoichiometry of Polδ subunits, which could be detrimental to Polδ folding and function.

 While we established that moderate over-expression of Polδ does not noticeably affect canonical replication, we acknowledge that presented data do not sufficiently disprove the natural occurrence of the stochastic switch from Polε to Polδ. Nevertheless, our data do imply that, if such events occur *in vivo*, they manifest at low frequencies and likely represent only a marginal disturbance to an overwhelmingly robust replication program.

## Data availability

### Underlying data

Gene Expression Omnibus: Raw and processed Pu-Seq data, Accession number GSE165503; https://identifiers.org/geo:GSE165503.

Zenodo: Increased expression of Polδ does not alter the canonical replication program in vivo. https://doi.org/10.5281/zenodo.4513956.

This project contains the following underlying data:

- Data_cell-size.xlsx (cell size measurements)
- data_OD.xlsx (optical density measurements)
- data_RT-qPCR.xlsx (raw Cq values)
- .tif files (raw microscopy images; strain-repeat-channel-image)
- .fcs files (flow cytometry files; well-strain-repeat-timepoint)

Data are available under the terms of the Creative Commons Attribution 4.0 International license (CC-BY 4.0).

## Software availability

Source code available from: https://github.com/R-Zach/Pu-Seq_polymerase_delta_over-expression

Archived source code at time of publication: https://doi.org/10.5281/zenodo.4516546 ( Zach, 2021)

License: Apache License 2.0
