# Supplementary material for: Increased expression of Polδ does not alter the canonical replication program in vivo
Source: Wellcome Open Res. 2021 May 4;6:44. Originally published 2021 Feb 26. [Version 2] doi: 10.12688/wellcomeopenres.16600.2 (PMC7974630; doi:10.12688/wellcomeopenres.16600.2)
Supplement: Peer review history

# Peer review: Reviewer response for version 2

**DOI:** 10.21956/wellcomeopenres.18586.r43673

**Published:** 2021-05-06

**Authors:** Jie Ren

**Affiliations:** 1 CAS Key Laboratory of Genomic and Precision Medicine, Beijing Institute of Genomics, Chinese Academy of Sciences, Beijing, China

No new comments.

Is the work clearly and accurately presented and does it cite the current literature?

Yes

If applicable, is the statistical analysis and its interpretation appropriate?

Yes

Are all the source data underlying the results available to ensure full reproducibility?

Yes

Is the study design appropriate and is the work technically sound?

Yes

Are the conclusions drawn adequately supported by the results?

Yes

Are sufficient details of methods and analysis provided to allow replication by others?

Yes

Reviewer Expertise:

Molecular genetics.

I confirm that I have read this submission and believe that I have an appropriate level of expertise to confirm that it is of an acceptable scientific standard.

# Peer review: Reviewer response for version 1

**DOI:** 10.21956/wellcomeopenres.18297.r42830

**Published:** 2021-03-18

**Authors:** Sarah Sabatinos

**Affiliations:** 1 Department of Chemistry and Biology, Ryerson University, Toronto, Canada

Zach and Carr examine the effect of polymerase delta over-expression on fission yeast DNA replication. This study tests whether the *in vitro* results of a 2017 study (Yeeles *et al.*) are found in fission yeast DNA replication, *in vivo*. Yeeles (2017) found that increasing polymerase delta concentration caused a decrease in synthesis, attributed to polymerase competition and leading strand polymerase switch.

 Zach and Carr have generated fission yeast strains that express additional copies of the 4 polymerase delta genes so that there are either 2 total copies or 4 total copies present in the genome. This design parallels the Yeeles *in vitro* work that similarly compared a 2x and 4x increase in polymerase delta. 

 The authors do not have antibody recognizing their polymerase delta proteins, but instead show that transcript levels of all 4 genes scale by qPCR. They characterize the effect of 2x, and 4x polymerase delta expression for its effects on: cell morphology, growth/doubling, and DNA content/cell cycle progression. Their calculation of growth uses absorbance readings, and is accompanied by cell length measurements to show that absorbance increase is not attributable to altered cell size. All results suggest that 2x and 4x increases in polymerase delta genes have no apparent effect on fission yeast cell fitness. Further, this over expression model does not noticeably slow DNA synthesis or cause replication-dependent effects on cell health and growth.

 The methods are clearly written, and provide thorough descriptions of materials and equipment used. Interestingly, the authors used 2 separate methods for Pu-Seq library preparation. The impact of using 2 different methods is not described in the text, and data from 1 experiment is shown in Figure 1. The "...Means of two independent experiments are shown." in figure 3, and the data suggests that differences between prepared libraries is minimal. Indication of replicates would be helpful if not explicitly stated (elaborated below).

 Overall, the authors provide a clear test of a specific hypothesis: that increased polymerase delta expression will impede synthesis. The data is clean, the premise is interesting, and the subject is worthy of study. The issue of not seeing protein level to characterize polymerase delta levels is minor in this initial *in vivo *characterization, given the characterization of transcript levels.

Minor comments:

- The methods are well-described and clear. Some elaboration of replicates would improve the text to be very clear throughout. Could experimental replicate number be indicated for 2C? Figure 3 suggests that the means of the 2 separate Pu-Seq methods are very close- is this the case? Are both Pu-Seq library replicates used in Figure 4 calculations?
- If polymerase delta switching to the leading strand impacts replisome speed, could this be seen in polymerase epsilon tracks? Figure 3 suggests that while the patterns are very similar for Pol e under over expression conditions, the overlap may not be perfect; in contrast, there is apparently tight overlay for normal/2x/4x in polymerase delta and alpha tracks. Is a non-perfect overlap in polymerase epsilon tracks the residual effect of a transient situation? Since Figure 3 shows the mean of 2 independent experiments, is the variation lost by combining individual experiments (prepared differently)?
- I have no complaint with the use of qPCR to quantify expression of the polymerases delta components, and transcript levels appear to scale by ~2x and ~4x relative to the baseline amount of each, independent transcript. Based on known promoter activities, how does the authors' 2x or 4x level of transcript increase (from amplified copies driven by the native promoters) compare to ectopic promoter-driven expression from MacNeill (1996), Kang (2000) or Reynolds (1998)? 
- How do efficient origins of replication in this work (Figure 4A) compare with other published studies?

Is the work clearly and accurately presented and does it cite the current literature?

Yes

If applicable, is the statistical analysis and its interpretation appropriate?

Yes

Are all the source data underlying the results available to ensure full reproducibility?

Yes

Is the study design appropriate and is the work technically sound?

Yes

Are the conclusions drawn adequately supported by the results?

Yes

Are sufficient details of methods and analysis provided to allow replication by others?

Yes

Reviewer Expertise:

Cell biology, molecular genetics.

I confirm that I have read this submission and believe that I have an appropriate level of expertise to confirm that it is of an acceptable scientific standard.

# Response

**Published:** 2021-04-19

**Authors:** Antony Carr

**Affiliations:** MRC Genome Damage and Stability Centre, University of Sussex, UK

- **The methods are well-described and clear. Some elaboration of replicates would improve the text to be very clear throughout. Could experimental replicate number be indicated for 2C? Figure 3 suggests that the means of the 2 separate Pu-Seq methods are very close- is this the case? Are both Pu-Seq library replicates used in Figure 4 calculations?**

In Figure 2C description, we included the number of independent measurements for WT, 2×Polδ and 4×Polδ cells. In Figure 1, Pu-Seq data from both experiments are shown now so reader can judge the variance of two methods used. In Figure 4, means of two experiments are analysed. This is now clearly stated in the Figure 4 description.

  

- **If polymerase delta switching to the leading strand impacts replisome speed, could this be seen in polymerase epsilon tracks? Figure 3 suggests that while the patterns are very similar for Pol e under over expression conditions, the overlap may not be perfect; in contrast, there is apparently tight overlay for normal/2x/4x in polymerase delta and alpha tracks. Is a non-perfect overlap in polymerase epsilon tracks the residual effect of a transient situation? Since Figure 3 shows the mean of 2 independent experiments, is the variation lost by combining individual experiments (prepared differently)?**

We argued that, if replisome speed was affected, we would detect changes in origin firing. Origins efficiency profiles in WT and Polδ-overexpressing cells; however, are comparable (Figure 4 – C, D). It is true that, according to Figure 3, Polε tracks in WT and Polδ-overexpressing cells do not display perfect overlap. We believe this is due to slightly higher inter-experimental variability in WT Polε datasets, as is now indicated in revised Figure 1.

  

- **I have no complaint with the use of qPCR to quantify expression of the polymerases delta components, and transcript levels appear to scale by ~2x and ~4x relative to the baseline amount of each, independent transcript. Based on known promoter activities, how does the authors' 2x or 4x level of transcript increase (from amplified copies driven by the native promoters) compare to ectopic promoter-driven expression from MacNeill (1996), Kang (2000) or Reynolds (1998)?**

In all listed studies, ectopic over-expression of non-catalytic Polδ subunits is inferred indirectly, based on a positive effect on cellular fitness of various replication mutants including *cdc1-P13*, *cdc6-121*, *cdc27-P11*, *cdc24-M38* and ﻿ *dna2-C2*. Successful over-expression of Cdc6 was judged by the fact that cells carrying a *nmt1-cdc6* construct developed severe cellular defects. Expression levels of Polδ subunits in different over-expression systems have never been demonstrated, so we cannot make a meaningful comparison.

- **How do efficient origins of replication in this work (Figure 4A) compare with other published studies?**

The original study by Daigaku *et al*. concluded that > 90% of origins identified by conventional method are also identified by Pu-Seq. Pu-Seq; however, provides better resolution and accuracy.

# Peer review: Reviewer response for version 1

**DOI:** 10.21956/wellcomeopenres.18297.r42833

**Published:** 2021-03-09

**Authors:** Jie Ren

**Affiliations:** 1 CAS Key Laboratory of Genomic and Precision Medicine, Beijing Institute of Genomics, Chinese Academy of Sciences, Beijing, China

This manuscript by Róbert Zach and Antony M. Carr examines the *in vivo *impact of an increased expression of DNA polymerase Polδ on cellular growth and replication dynamics.

 Intriguingly, although the canonical model divides the workload of leading strand synthesis and lagging strand synthesis between Polε and Polδ, *in vitro* and *in vivo* studies showed evidence of Polδ activity on both strands under certain circumstances. Yet, *in vitro* reconstitution of replication indicated that although increased dosage of Polδ can out compete Polε on the leading strand, this is much less efficient for its synthesis. The current work tests the hypothesis that whether the same effect exist *in vivo*, which will provide more understanding on replication dynamics in living cells.

 Using a carefully designed Cre-Lox system, the authors constructed strains with two- or four-fold increased Polδ holoenzyme with the ability to incorporate rNTPs for following assessment of polymerase usage. With various analysis on cell growth and genome-wide quantification of individual polymerase usage especially at origins and termination regions, they concluded that there is no detectable impact in replication dynamics or associated growth defect caused by increased amounts of Polδ.

 Major Comments:

1. It will be nice to also show an example for termination site identification through Pu-seq as the demonstration for origin in Fig 1.
2. In the method section, the authors mentioned that an independent set of modified GLOE-seq was also performed along side Pu-seq. Are the GLOE-seq results comparable with those of Pu-seq? Is this work still undergoing?
3. Increased amount of Polδ transcripts were confirmed with the over-expression system, but increased usage of Polδ is barely evident as shown by this work. Although not required for the current work, it will be nice to be followed up by testing whether or not more Polδ subunits can be incorporated into the replisome using iPOND or similar techniques.

Minor points:

1. In the analysis of Pu-seq data, PT is calculated as (Rt+Rb)/(Rt-Rb). Is there certain mathematical conversion omitted here? Otherwise, how is the range of PT kept within [-1,1] as in Fig 1?
2. It is concluded that Polα tracks in 2xPolδ is marginally different from the other two conditions. I assume it is demonstrated by the mean polymerase tracks in Fig 4A and 4B as a slight deviation of the curve of squares from the other two curves. It is better to make that clear in the figure legend, or with some statistical analysis.

Is the work clearly and accurately presented and does it cite the current literature?

Yes

If applicable, is the statistical analysis and its interpretation appropriate?

Yes

Are all the source data underlying the results available to ensure full reproducibility?

Yes

Is the study design appropriate and is the work technically sound?

Yes

Are the conclusions drawn adequately supported by the results?

Yes

Are sufficient details of methods and analysis provided to allow replication by others?

Yes

Reviewer Expertise:

Molecular genetics.

I confirm that I have read this submission and believe that I have an appropriate level of expertise to confirm that it is of an acceptable scientific standard.

# Response

**Published:** 2021-04-19

**Authors:** Antony Carr

**Affiliations:** MRC Genome Damage and Stability Centre, University of Sussex, UK

**Major Comments:**

  

- **It will be nice to also show an example for termination site identification through Pu-seq as the demonstration for origin in Fig 1.**

Representative locus in Figure 1 was expanded. Now the origin of replication as well as the adjacent termination zone are displayed. Additionally, data from both experiments are shown.

  

- **In the method section, the authors mentioned that an independent set of modified GLOE- seq was also performed alongside Pu-seq. Are the GLOE-seq results comparable with those of Pu-seq? Is this work still undergoing?**

Modified GLOE-Seq protocol was used to prepare the second set of Pu-Seq libraries (experiment/repeat 2). Figure 1 now shows Pu-Seq data generated by both procedures.

  

- **Increased amount of Polδ transcripts were confirmed with the over-expression system, but increased usage of Polδ is barely evident as shown by this work. Although not required for the current work, it will be nice to be followed up by testing whether or not more Polδ subunits can be incorporated into the replisome using iPOND or similar techniques. **

This is a valuable suggestion. It indeed would be interesting to investigate how replisome composition reacts to different levels of its particular components, such as Polδ.

**Minor points:**

- **In the analysis of Pu-seq data, PT is calculated as (Rt+Rb)/(Rt-Rb). Is there certain mathematical conversion omitted here? Otherwise, how is the range of PT kept within [-1,1] as in Fig 1?**

In the manuscript we made a mistake and stated PT = (R _T_ + R _B_) / (R _T_ – R _B_) instead of PT = (R _T_ – R _B_) / (R _T_ + R _B_), which is the correct equation used in the analysis. Typo was corrected. The fact that denominator is always bigger than nominator ensures that PT falls within [-1,1].

- **It is concluded that Polα tracks in 2xPolδ is marginally different from the other two conditions. I assume it is demonstrated by the mean polymerase tracks in Fig 4A and 4B as a slight deviation of the curve of squares from the other two curves. It is better to make that clear in the figure legend, or with some statistical analysis.**

Minor deviations in Polα tracks are now signified by asterisks.

**Additional changes to the manuscript:**

- In the introduction section, we now reference a recent paper discussing the role of Polδ in bypassing oxidative DNA lesions:

*“Additionally, it has been demonstrated that Polδ takes over the leading strand synthesis and performs an error-free bypass of oxidative DNA adducts thymine glycol and 8-oxoguanine (Guilliam & Yeeles, 2021).”*
